# Variability in Functional Traits along an Environmental Gradient in the South African Resurrection Plant *Myrothamnus flabellifolia*

**DOI:** 10.3390/plants11101332

**Published:** 2022-05-18

**Authors:** Rose A. Marks, Mpho Mbobe, Marilize Greyling, Jennie Pretorius, David Nicholas McLetchie, Robert VanBuren, Jill M. Farrant

**Affiliations:** 1 Department of Horticulture, Michigan State University, East Lansing, MI 48824, USA; vanbur31@msu.edu; 2 Plant Resilience Institute, Michigan State University, East Lansing, MI 48824, USA; 3 Department of Molecular and Cell Biology, Faculty of Science, University of Cape Town, Cape Town 7700, South Africa; jill.farrant@uct.ac.za; 4 Welgevonden Environmental Awareness Program, Vaalwater 0530, South Africa; mphoceline27@gmail.com; 5 Waterberg Research Support Center, Vaalwater 0530, South Africa; info@waterbergresearch.org.za; 6 Buffelskloof Nature Preserve, Lydenburg 1120, South Africa; trailsafari@gmail.com; 7 Department of Biology, University of Kentucky, Lexington, KY 40506, USA; mclet@uky.edu

**Keywords:** desiccation tolerance, abiotic stress, drought, extremophyte, *Myrothamnus flabellifolia*, resurrection plant, South Africa, tradeoffs

## Abstract

Many desiccation-tolerant plants are widely distributed and exposed to substantial environmental variation across their native range. These environmental differences generate site-specific selective pressures that could drive natural variation in desiccation tolerance across populations. If identified, such natural variation can be used to target tolerance-enhancing characteristics and identify trait associations within a common genetic background. Here, we tested for natural variation in desiccation tolerance across wild populations of the South African resurrection plant *Myrothamnus flabellifolia*. We surveyed a suite of functional traits related to desiccation tolerance, leaf economics, and reproductive allocation in *M. flabellifolia* to test for trait associations and tradeoffs. Despite considerable environmental variation across the study area, *M. flabellifolia* plants were extremely desiccation tolerant at all sites, suggesting that tolerance is either maintained by selection or fixed in these populations. However, we detected notable associations between environmental variation, population characteristics, and fitness traits. Relative to mesic sites, plants in xeric sites were more abundant and larger, but were slower growing and less reproductive. The negative association between growth and reproduction with plant size and abundance pointed towards a potential growth–abundance tradeoff. The finding that *M. flabellifolia* is more common in xeric sites despite reductions in growth rate and reproduction suggests that these plants thrive in extreme aridity.

## 1. Introduction

The diverse adaptations displayed by plants comprise a valuable reservoir of biological information that can be leveraged to develop solutions for agricultural challenges, inform conservation practices, and provide value to local communities. However, the preservation and utilization of these botanical resources hinges on a comprehensive understanding of their ecology and natural diversity so that informed land management and conservation decisions can be made [1]. Understanding the biology of plants adapted to marginal and extreme environments is particularly relevant in the face of climate change [2]. The genetic information encoded in stress-adapted species could be used to improve resilience in other species via gene editing and bioengineering approaches, and insight into the ecology of these plants can be used to maximize sustainability and improve predictions of species responses to climate change. However, because stress tolerance traits did not evolve in static environments or under uniform selective pressures, the mechanisms and ecology of stress adaptation are extremely complex and variable [3,4,5,6,7], and many questions remain.

Resurrection plants are, in many ways, the epitome of stress tolerant plants and are an ideal system for untangling mechanisms of tolerance. Resurrection plants are a phylogenetically diverse group of plants that occur in extremely arid habitats around the world [8,9,10,11,12,13,14]. Resurrection plants can tolerate nearly complete desiccation of their vegetative tissues—to or below an absolute water content of −100 MPa—without dying [15]. In fact, most resurrection plants can persist in a desiccated state for months to years, during which time they may be exposed to intense heat and irradiation, yet they still recover normal photosynthetic and metabolic processes within hours of the first rains [16,17]. Desiccation tolerance has evolved recurrently across diverse plant lineages spanning over 500 million years of divergence, and the mechanisms used to attain tolerance vary considerably across species [3,4,5,6,7]. Tolerance can also vary within the vegetative tissues of a single individual [18,19] as can other functional traits, but variation in desiccation tolerance across natural populations of resurrection plants has been underexplored. Many resurrection plants are exposed to notable environmental differences across their native range that could generate site-specific selective pressures, drive local adaptation, and lead to intraspecific variation among populations [20,21]. However, some species have maintained desiccation tolerance despite being restricted to mesic habitats, suggesting that desiccation tolerance may be less labile than other traits. For example, *Lindernia brevidens* is endemic to montane forests in Tanzania and Kenya where it rarely (if ever) experiences desiccation, yet still displays desiccation tolerance [22]. If detectable, within species, natural variation would provide a valuable opportunity to identify tolerance enhancing characteristics and genes, describe positive and negative tradeoffs, and enhance ecological predictions, while minimizing the background differences that complicate cross-species comparisons. 

Here, we characterized the natural variation in desiccation tolerance, abundance, and fitness traits of the resurrection plant *Myrothamnus flabellifolia* across an environmental gradient in South Africa. Although *M. flabellifolia* plants are generally restricted to sites where abiotic stresses (e.g., heat, irradiation, drought) are high, notable environmental variation is evident across the species’ native range [23,24,25]. We hypothesized that plants occurring in the most xeric sites would have elevated desiccation tolerance relative to plants in more mesic sites; that abundance and productivity traits would scale with increasing rainfall; and that desiccation tolerance would tradeoff with growth and reproduction, as outlined in CSR (competitor, stress-tolerator, ruderal) theory [26]. However, we also predicted that stress tolerance traits would exhibit less intraspecific variability than growth and reproductive traits due to selection on desiccation tolerance even in the more mesic sites (because water limitation still occurs there). 

Sexual dimorphisms in stress tolerance add another layer to understanding population dynamics in diecious species. These dimorphisms can differentially impact the survival of male and female plants, drive spatial segregation of the sexes, and lead to biased population sex ratios [27,28]. In most angiosperms, females are expected to be less stress tolerant than males due to increased demand for water and carbon during the reproductive processes of fruit and seed maturation [28,29]. Thus, females are expected to perform worse under drought conditions and be less abundant in drought-prone sites. However, others have argued that females may experience selection to persist under drought conditions in order to mature offspring. How these contrasting pressures interact to influence whole organism responses to water shortage is unclear, and empirical evidence on the topic is contradictory [30,31,32]. To better understand the consequences of sexual dimorphisms in stress tolerance, we quantified sex-specific functional traits in *M. flabellifolia* and assessed population sex ratios.

*Myrothamnus flabellifolia* is an important resurrection plant that stands at the intersection of multiple cultures and diverse stakeholders. The species is native to southern Africa and has been independently named in multiple languages. It is known as *Uvukakwabafile* in isiZulu, *Patje-ya-tshwene* in Setswana/Sesotho, *Umazifisi* in isiNdebele, and *Mufandichumuka* in Shona. The plants produce a robust profile of secondary compounds related to tolerance and defense [33], many of which have important historical and contemporary medicinal applications [25,33,34,35]). Traditional healers in southern Africa use the plant as a medicinal tea to improve the quality of sleep, cure common colds, mitigate fatigue and stress, and cure infertility. Sometimes, the leaves are burned, and the smoke is used to treat respiratory ailments such as asthma, headaches, and nosebleeds; to revive people who have fainted; and to treat uterine pain (personal communication MM and RAM). More recently, the plant has been used to treat cancer, reduce blood pressure, and prevent kidney damage [35,36]. In addition to traditional and medicinal uses, there is growing international interest in *M. flabellifolia* for cosmetic and pharmaceutical applications, and multiple international companies are exploring *M. flabellifolia* extracts for product development. Without ecologically informed management, these competing interests could lead to over harvest and population decline [36]. The work presented here can be used to inform management and conservation decisions regarding *M. flabellifolia.*

## 2. Materials and Methods

### 2.1. Study Organism 

**Figure 1:**
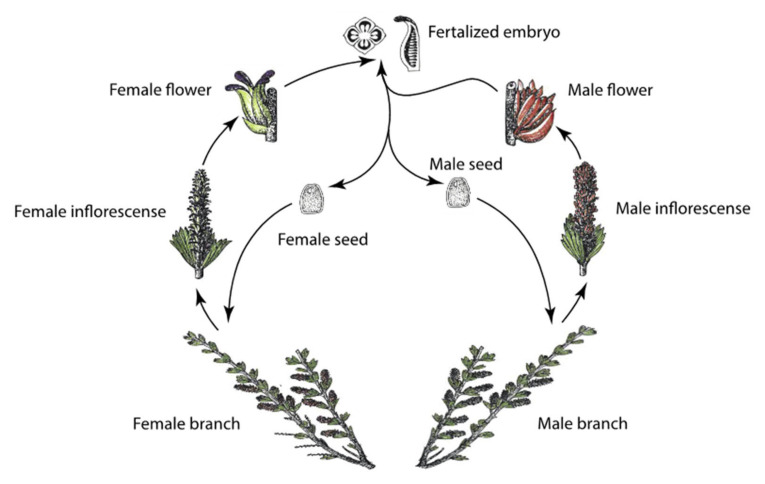
Life cycle and anatomy of *Myrothamnus flabellifolia***.** Plants are dioecious and produce sex-specific inflorescences. Vegetative tissue, flowers, and seeds are desiccation tolerant.

*Myrothamnus flabellifolia* is a resurrection plant in the eudicot order Gunnerales. Plants are distributed throughout southern Africa in disjunct populations from Namibia to Tanzania, with the highest density of plants occurring in South Africa and Zimbabwe [24,37]. *Myrothamnus flabellifolia* is a woody shrub growing up to ≈1.5 m tall with highly branched anatomy, short internode length (≈0.5–1.5 cm), and opposite leaf arrangement. Leaves are small, with short petioles and parallel venation. *Myrothamnus flabellifolia* is dioecious, and inflorescences consist of densely packed florets in a simple arrangement with extremely short pedicles. Male inflorescences have bracts, but female inflorescences are bract-less (Figure 1).

### 2.2. Study Area and Environmental Characteristics

**Figure 2:**
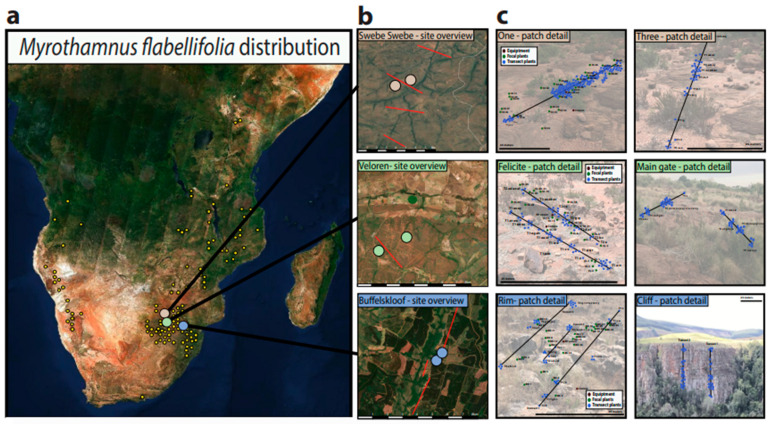
Overview of the study area and sampling design. (**a**) The distribution of *Myrothamnus flabellifolia* is visualized from data available at www.gbif.org (accessed on 15 September 2020). (**b**) The three study sites are located in northeastern South Africa and are within 400 km of one another. Red lines show the location of large mapping transects. (**c**) Within each site, two patches of *M. flabellifolia* were sampled in detail, and phenotypic data on plants (blue dots) were recorded along sampling transects within each patch. At select patches, we tagged 25 focal plants (green dots) for detailed measurements and long-term monitoring. Background images of patches are representative photos of each patch and are not scaled to size.

For the current work, three study sites were established in north-eastern South Africa: Buffelskloof Nature Reserve in Mpumalanga province (−25.30229 S, 030.50631 E); Veloren Wildlife Estate (−24.7863 S, 028.3755 E); and Swebe Swebe Nature Reserve (−23.7949 S, 028.0705 E) in the Waterberg, Limpopo province (Figure 2). These sites span 400 km and were selected to capture the maximal environmental variation possible within the study area. Historical weather data from 1981 to 2019 on temperature, precipitation, and relative humidity was downloaded from NASA’s Prediction of Worldwide Energy Resources platform (https://power.larc.nasa.gov, accessed on 1 September 2020) using the GPS coordinates for the center of each site. These data were analyzed to test for differences in annual temperature, rainfall, and relative humidity across the three study sites. Site-specific elevations were recorded using a Garmin 64csx GPS, bedrock types were visually assessed and cross referenced with geological records, and soil depths were measured at the base of every plant (≈50–200 per site).

### 2.3. Habitat Characteristics and Plant Sampling

Sampling was conducted during the rainy season in north-eastern South Africa (~November to March) for three consecutive years (2019–2022). Initially, we estimated the overall abundance of *M. flabellifolia* at each of the study sites via large mapping transects. Transects were established, bisecting each site at regular intervals, and we sampled every *M. flabellifolia* patch encountered along these transects. We recorded the area, elevation, slope, aspect, and density of plants in each patch. In addition, the sex of all plants long the central access of the patch was recorded, and the dominant co-occurring species were documented. We used these data to estimate the abundance, density, and sex ratios of *M. flabellifolaia* populations. Population sex ratios were calculated as number of males relative to the total number of reproductive individuals.

Subsequently, functional traits were measured within a single focal patch at each site. We targeted patches that were at least 100 m away from the nearest road to minimize disturbance and where *M. flabellifolia* was among the dominant species. Within each patch, we established transects spanning a cumulative distance of ≈60–100 m. The first transect was set to span the longest axis of the patch and subsequent transects were established until a cumulative distance of 60 m or more was sampled. We sampled all plants that occurred within one meter on either side of each transect for a total of ≈50–150 plants per patch. We targeted plants that appeared to be separate genets with no above ground connections. One of the patches at Buffelskloof is on a vertical cliff. There, we established vertical transects by abseiling over the cliff and sampling plants as described above.

In addition to sampling plants along transects, we selected 25 focal plants within each patch for the most detailed phenotypic measurements. Here, we intentionally targeted plants that were medium size (≈30–80 cm tall), healthy, and growing in full sun with minimal evidence of shading or competition. These plants were tagged for long term monitoring and used for measures of desiccation tolerance, growth rate, flowering, architecture, and specific leaf area (SLA). Specimens were vouchered at the Buffelskloof Nature Reserve Herbarium, Lydenberg, Mpumalanga, South Africa (specimen number BNRH0025621).

### 2.4. Desiccation Tolerance Traits

We quantified three traits related to desiccation stress and recovery. First, we estimated the intensity of a typical drying event at each site by assessing the relative water content (RWC) of field dry material for the set of 25 focal plants per site. We collected a single terminal twig (≈10 cm long) from each plant in a desiccated state. The desiccated leaves were removed from the twig and their mass (fresh mass) was determined immediately after collection using a Frankford Arsenal DS-750 scale. Leaf tissue was then fully submerged in water and placed at 4 °C in complete darkness for 48 h, after which tissues were blotted dry and their turgid mass was assessed. Tissues were then transported to the University of Cape Town and dried at 70 °C for 2 days in a drying oven, and then their dry mass was measured. RWC was calculated as [(fresh mass-dry mass)/(turgid mass-dry mass) × 100].

Next, we assessed rehydration dynamics and recovery outcomes of field desiccated plants. To do so, three randomly selected terminal twigs (≈6–13 cm) were cut from each of the 25 focal plants per site in a desiccated state. Twigs were photographed immediately upon collection and then placed in individual 50 mL falcon tubes containing 15 mL water each and allowed to rehydrate in ambient conditions. Plants were imaged again after 2, 4, 8, 12, and 24 h of rehydration. From these images, we computed the percent of recovered tissue for each twig (25 individuals per site, 3 replicates per individual, totaling 225 twigs across all three sites) in ImageJ v1.53 [38]. We scored each leaf as recovered or not, on the basis of its color and shape. Briefly, leaves that were fully expanded and green were scored as “recovered”, whereas leaves that were folded and/or brown/yellow were scored as “not recovered”. We used this binary scale to assess each individual leaf and then computed the percentage of recovered leaves on each twig. We used these data to estimate the rate of rehydration throughout the photographic time-series and to quantify the final recovery outcome.

### 2.5. Growth and Vegetative Traits

We investigated a suite of vegetative traits. First, we quantified plant height by measuring the distance from the soil to the top of the tallest branch. Growth rate was estimated for the 25 focal plants per site using three randomly selected terminal twigs per plant. In year one, we marked each twig 10 cm from the tip. In each subsequent year, we measured the distance from that mark to the tip of the twig. Any distance beyond 10 cm was considered new growth. Compactness (leaf frequency) was estimated for the 25 focal plants per site using photographic data from the rehydration time-series described above. Briefly, we counted the number of leaf pairs on each twig using ImageJ v1.53 [38] and divided that by the total length of the twig to determine the number of leaves per cm. Leaf area was measured using images of 5–10 fully hydrated leaves per plant in ImageJ v1.53. Those leaves were then dried at 70 °C for 2 days and their dry mass was assessed. From these measures, we computed SLA as (leaf area/dry mass).

### 2.6. Reproductive Traits

Male and female *M. flabellifolia* plants have distinct floral morphology (Figure 1), and sex was determined visually when plants were in flower. To investigate differences in reproductive allocation across the sites and sexes, we estimated the number of inflorescences produced by each plant at the beginning of the rainy season in 2019 and again in 2021. Because *M. flabellifolia* can produce dozens of inflorescences on a single plant, we subsampled plants by randomly selecting three apical branches on each plant and counted the number of inflorescences on the top 10 cm of each branch (*n* = 525).

### 2.7. Statistical Analyses

First, we tested for environmental differences across sites (e.g., rainfall, temperature, relative humidity, and soil depth) using mixed effects linear models with year included as a random effect. Next, we tested the effect of site, sex, and their interaction on RWC using a mixed effects linear model. Plant ID and twig replicate were included as random effects. Percent recovery data were log transformed to improve normality, and the effects of site, sex, and their interaction were tested using a mixed effect linear model. Plant ID and twig replicate were included as random effects. To understand the rate of rehydration, we used MANOVA repeated measures analysis to test the effect of site and sex on the proportion of leaves open over time. For plant height, growth rate, compactness, SLA, and the number of inflorescences produced, we used mixed effects linear models to test the effects of site, sex, and their interaction on each response variable, individually. Plant ID and twig replicate were included as random effects where applicable. Contrasts were used to test for differences between each pairwise combination of sites and the sexes. A heterogeneity test was used to test if population sex ratios were different from one another, and a goodness of fit test was used to identify sex ratios significantly different from 50:50.

To better understand trait relationships, tradeoffs, and associations, we conducted dimension reduction analyses on all vegetative and reproductive traits. These analyses were performed on the set of 25 focal plants per site and included trait values for height, growth rate, flower production, RWC, percent recovery, compactness, leaf area, and SLA. First, we generated correlation matrices for each pairwise combination of traits to identify the most positively and negatively associated traits. Next, we conducted principal component analysis (PCA) to visualize sample and trait relationships.

All data associated with this study are provided as Supplementary Material (Table S1).

## 3. Results

### 3.1. Habitat Characteristics

**Figure 3:**
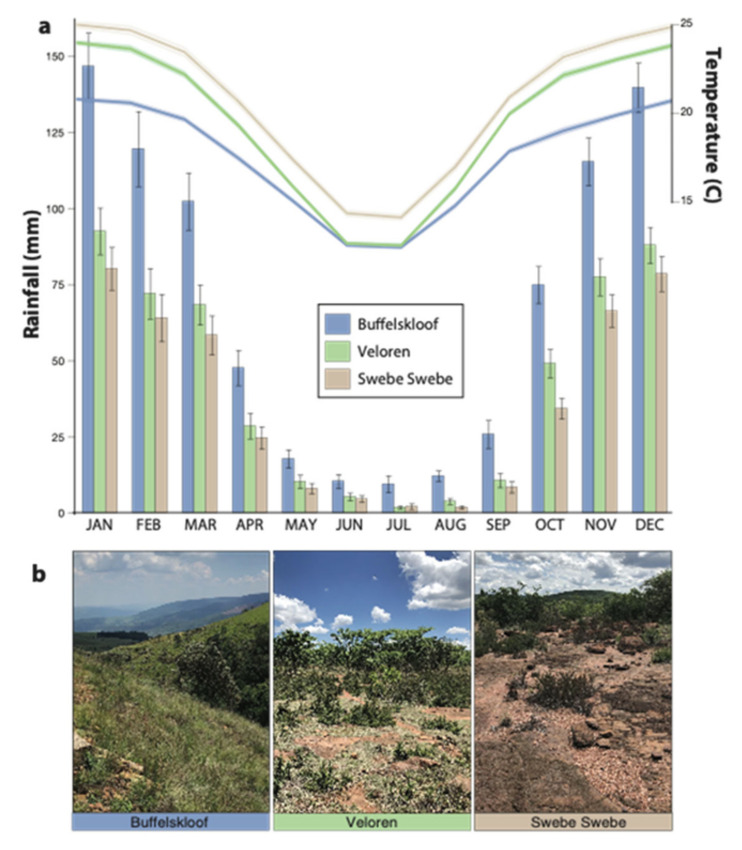
Environmental conditions at the three study sites, ordered from wettest to driest. (**a**) Differences in mean monthly temperature (lines) and rainfall (bars) were evident across sites. (**b**) All three sites were classified as dry winter climates, but differences in vegetative cover and community composition were evident.

Noticeable environmental differences were detected across the study sites (Figure 3a). Buffelskloof is the wettest, coolest, and highest elevation site; Veloren is intermediate on all three measures; and Swebe Swebe is the driest, hottest, and lowest elevation site. Annual rainfall differed significantly (F_2,113_ = 52.1, *p* < 0.0001), ranging from 820 ± 41.5 mm at Buffelskloof (the wettest site), to 507 ± 26.3 mm at Veloren (the intermediate site), and 430 ± 23.1 mm at Swebe Swebe (the driest site). Significant differences in mean annual relative humidity (F_2,113_ = 204.2, *p* < 0.0001) and temperature (F_2,113_ = 289.7, *p* < 0.0001) were also detected along a similar gradient. Relative humidity ranged from 61 ± 0.7% at Buffelskloof, to 47 ± 0.6% at Veloren, and 45 ± 0.6% at Swebe Swebe. Temperatures ranged from 18 ± 0.1 °C at Buffelskloof, to 20 ± 0.1 °C at Veloren, and 21 ± 0.1 °C at Swebe Swebe. These sites also occur on different bedrock types. Buffelskloof is situated on quartzite at 1492 m altitude, Veloren on sandstone at 1334 M, and Swebe Swebe on conglomerate sandstone at 1068 m. The various substrates can contribute to differences in hydrology, mineral nutrient availability, and thermal dynamics, which further differentiate the ecological conditions across these sites. Soil depth was low across all sites, with mean depth ranging from 6.04 ± 0.35 cm at Buffelskloof, to 7.5 ± 0.38 cm at Veloren, and 6.13 ± 0.21 cm at Swebe Swebe, but differences across sites were significant (F_2,210_ = 6.79, *p* = 0.0014). Somewhat counterintuitively, the cliff population at Buffelskloof had the deepest soil depth, but this consisted of sporadic pockets of deep soil separated by vertical rock. Taken together, these variables combine to generate perceptibly different environmental conditions at the three study sites (Figure 3b).

### 3.2. Desiccation Tolerance Traits

**Figure 4:**
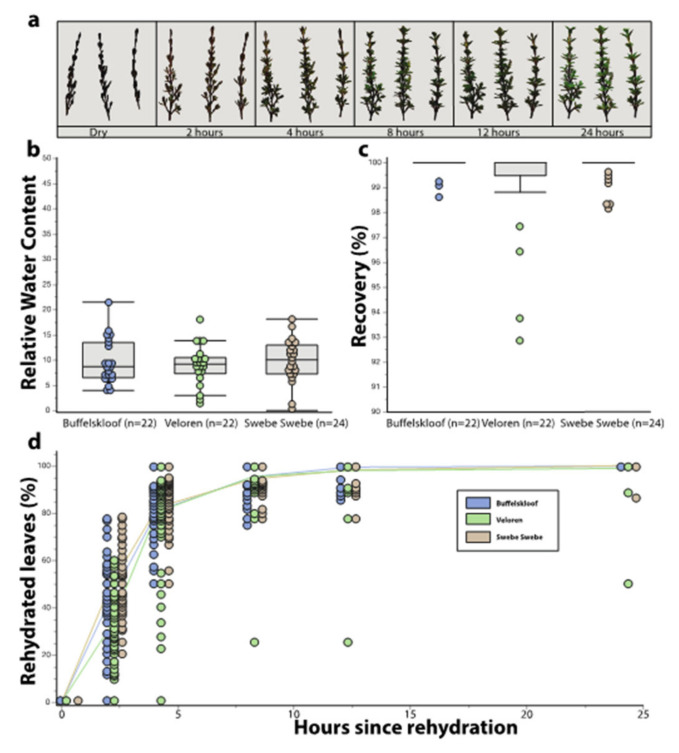
Stress tolerance traits across the three study sites, ordered from wettest to driest. (**a**) Pictorial representation of a rehydration time-series from one representative plant from Buffelskloof. These, and similar images for all plants, were used to estimate the proportion of recovered leaves at each timepoint and to assess percent recovery. (**b**) The relative water content of plants collected from the field in a desiccated state did not differ across the sites. (**c**) The percentage of leaves that recovered to a green and healthy condition within 24 h did not differ across the sites. (**d**) Plants from Veloren were significantly slower to rehydrate than plants from Swebe Swebe and Buffelskloof.

Desiccation tolerance traits exhibited minimal variation in *M. flabellifolia* plants across the three study sites. RWC of field desiccated plants was not significantly different. Plants had a mean RWC of 9.6 ± 0.9% at Buffelskloof, 9.0 ± 0.8% at Veloren, and 9.8 ± 0.9% at Swebe Swebe (Figure 4b). *Myrothamnus flabellifolia* tissues exhibited almost complete recovery across all three sites with mean percent recovery of 99.95 ± 0.03% at Buffelskloof, 99.53 ± 0.16% at Veloren, and 99.89 ± 0.05% at Swebe Swebe. There were no significant differences in recovery across these sites (Figure 4c). The rate of rehydration showed significant differences across sites (F_2,192_ = 2.35, *p* = 0.0021). This difference was only significant at early timepoints. Plants from Veloren were the slowest to rehydrate, and plants from Swebe Swebe were the fastest (Figure 4d).

### 3.3. Growth and Vegetative Traits

**Figure 5:**
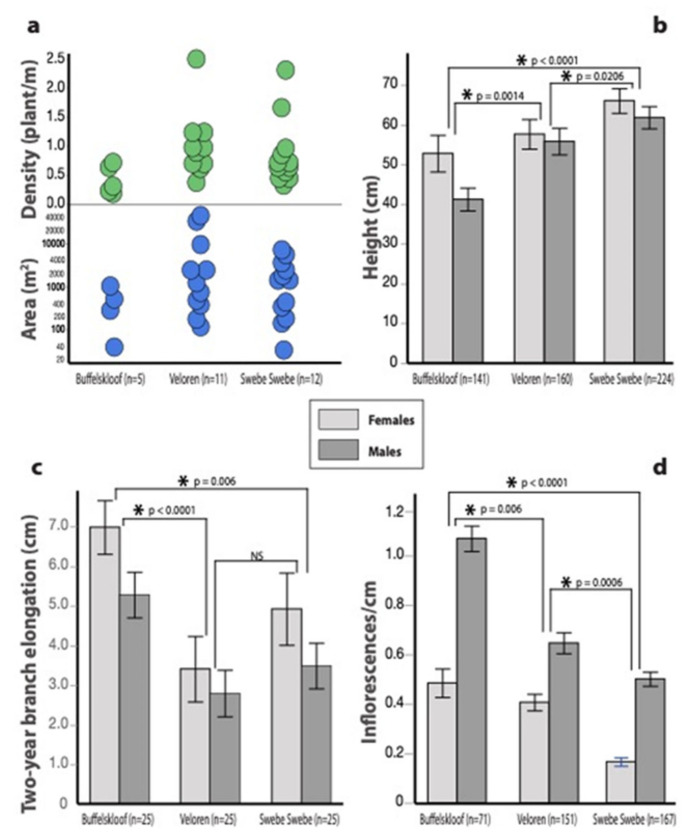
Vegetative and fitness traits across the three study sites, ordered from wettest to driest. (**a**) Area and size of patches at each site. (**b**) Plant height decreased with increasing water availability and altitude. Males were shorter than females. (**c**) Branch elongation (a proxy for growth rate) decreased with water availability and altitude. Females grew faster than males. (**d**) The number of inflorescences produced declined in parallel with water availability and elevation. Males produced more inflorescences than females.

Vegetative traits exhibited considerably more variability than desiccation tolerance traits. In general, patches of *M. flabellifolia* were more frequent, larger, and contained a higher density of plants at Veloren and Swebe Swebe (the more xeric sites) relative to Buffelskloof (the most mesic site) (Figure 5a). Significant differences across sites in plant height (F_2,262_ = 15.21, *p* < 0.0001) (Figure 5b), growth rate (F_2,202_ = 9.33, *p* = 0.0001) (Figure 5c), compactness (F_2,193_ = 6.11, *p* < 0.0001), and SLA (F_2,63_ = 3.24, *p* = 0.046) were identified. Plants at Buffelskloof were the shortest (35.3 ± 2.15 cm), those at Veloren were intermediate (45.9 ± 2.30 cm), and plants at Swebe Swebe were the tallest (55.7 ± 1.68 cm). Conversely, plants from Buffelskloof were the fastest growing (putting on 6.1 ± 0.45 cm of new growth every two years), whereas plants from Veloren and Swebe Swebe grew slower (putting on only 3.0 ± 0.48 and 4.1 ± 0.52 cm of growth every two years, respectively). Plants at Veloren were the most compact (1.4 ± 0.04 leaves per cm) and had the lowest SLA (8.9 ± 0.39), whereas those at Swebe Swebe were least compact (1.09 ± 0.03 leaves per cm) and had the highest SLA (9.43 ± 0.38). Plants from Buffelskloof were intermediate for both (1.28 ± 0.04 leaves per cm and 8.80 ± 0.26 SLA). 

### 3.4. Reproductive Traits

We identified significant differences in the number of inflorescences produced at each site (F_2,226_ = 19.9, *p* < 0.0001). Plants from Buffelskloof produced the most inflorescences (8.7 ± 0.44 inflorescences per 10 cm), plants from Veloren were intermediate (5.2 ± 0.27 inflorescences per 10 cm), and plants from Swebe Swebe produced the fewest (3.6 ± 0.19 inflorescences per 10 cm) (Figure 5d). Sexual dimorphisms are discussed below.

### 3.5. Sexual Dimorphisms

**Figure 6:**
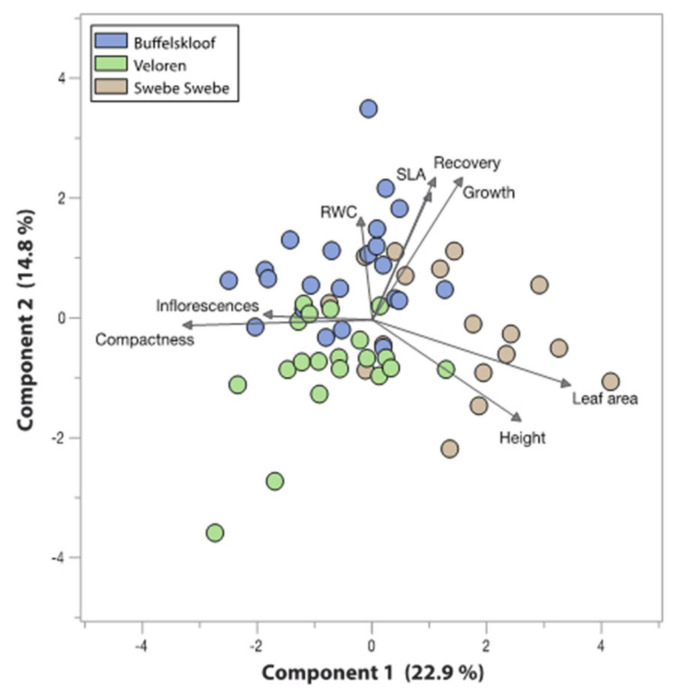
Principal component analysis of six traits for 25 focal plants per site. Trait projections are plotted on the graph as arrows and each plant is indicated by a single point. Points are colored by the study site.

We were able to determine the sex of a relatively high proportion of plants (≈70% in most sites) (Figure 6). The only exceptions were cliff-dwelling plants at Buffelskloof, which had much lower flowering rate, possibly due to limited light exposure. We did not detect sexual dimorphisms in any stress tolerance traits. Male and female plants had equivalent field RWC, rehydrated at the same rate, and had similarly high percentages of recovery. However, there were significant differences between the sexes in height, growth rate, and inflorescence production. Females were significantly taller (F_1,300_ = 3.9, *p* = 0.0479) and grew significantly faster (F_1,202_ = 5.1, *p* = 0.0255) than males. In contrast, males produced significantly more inflorescences than females (F_1,262_ = 84.5, *p* < 0.0001). We detected variable sex ratios across patches and sites. Buffelskloof was slightly (but not significantly) female biased, whereas Veloren and Swebe Swebe were significantly male biased (G_1_ = 3.8, *p* = 0.05 and G_1_ = 3.7, *p* = 0.05 respectively).

### 3.6. Trait Associations and Tradeoffs

The most positively associated traits –s in our study were height and leaf area (tall plants had large leaves). Our analyses also identified multiple negatively associated traits, which point towards possible tradeoffs. Height and compactness (taller plants were less compact), compactness and leaf area (more compact plants had smaller leaves), and leaf area and inflorescence number (plants with larger leaves produced fewer inflorescences) were all negatively associated (Table 1). PCA reinforced these associations, showing that height and leaf area project in the same direction and are opposed to compactness and inflorescence production (Figure 6). Plants from Swebe Swebe formed the most distinct cluster and were characterized by increased height and large leaf area with fewer inflorescences and less compact leaf arrangement.

## 4. Discussion

Our study provides one of the first estimates of natural variation within an angiosperm resurrection plant. We detected differences in local abundance, population characteristics, and vegetative traits across the study area. However, we observed minimal variability in desiccation tolerance, despite marked environmental differences between sites. *Myrothamnus flabellifolia* plants were extremely stress tolerant at all sites, suggesting that the trait is either maintained by selection even in more mesic areas—because drought does still occur there—or fixed. It is possible that reductions in desiccation tolerance could arise in more environmentally mesic habitats or are only evident on longer timescales (i.e., longevity in a dry state), but those factors were not tested here.

We detected complex and noteworthy phenotypic variation in vegetative, reproductive, and population traits across the study area. Plants from the most mesic study site (Buffelskloof) were fast growing and highly reproductive, but those populations were the smallest, least frequent, and comprised the shortest plants. In contrast, plants from Veloren exhibited slow growth rates and intermediate flowering, but were tall, extremely compact, and very abundant. Plants from the driest site (Swebe Swebe) were also abundant, with slow growth rates, reduced flowering, tall stature, and open architecture. Taken together, we found that despite being large and abundant, plants in the drier sites grow and reproduce slowly, indicating that these populations are unsuitable areas for harvest unless careful regulations are in place to prevent depletion of the populations. Conversely, plants from the more mesic site were faster growing and more reproductive, but maintained low population density and short stature, suggesting that these populations experience considerable mortality during establishment or competitive exclusion. Although we did not quantify competitive interactions directly, they is likely a larger factor at the mesic study site, because vegetative cover is much higher there. This could contribute to low establishment and even explain the high investment in sex as a potential escape mechanism. Alternatively, the small size of plants in the mesic site could be associated with high altitude, wind, herbivory, fire, or even “desiccation pruning”—a phenomenon in which plants self-prune because they are unable to refill their xylem above a certain height.

Identifying sexual dimorphisms is an important step to understanding how to conserve and protect dioecious plants [39]. Because females bare the cost of maturing offspring (i.e., spores, seeds, and fruits), they require more carbon and water resources during that developmental stage [40]. Therefore, females are expected to either suffer more under water limitation [41], or be selected for higher water stress tolerance. In the current study, we found a reduction of females in water limited habitats—the two driest sites were male biased whereas the more mesic site had a higher proportion of females—supporting the hypothesis that high demand for water can limit female success in increasingly dry environments. We identified multiple sexually dimorphic traits that could contribute to the observed sex ratio variation. Males produced more inflorescences than females at all sites, but females were taller and faster growing at all sites. This finding is not surprising, as males typically invest more in pre-fertilization reproductive processes, whereas females are expected to invest more in resource acquisition in order to accommodate their substantial post-fertilization reproductive costs [42,43,44]. Perhaps females outperform males in mesic areas because higher investment in growth gives them a competitive edge in these more productive sites. In the drier sites, both growth and reproductive allocation are reduced, and this appears to shift the competitive advantage to males. Taken together, these findings suggest that although desiccation tolerance is not dimorphic in *M. flabellifolia*, secondary sexual dimorphisms in growth and reproduction could drive sex ratio variation along an environmental gradient.

In conclusion, we show that desiccation tolerance is extremely conserved in *M. flabellifolia*. Despite considerable environmental variation, plants were extremely and similarly stress tolerant at all sites. However, we detected notable variation in other life history and morphological traits, suggesting that these plants are finely tuned to their environment. We show that plants from the most mesic sites are more reproductive and faster growing, whereas plants from the more xeric sites are larger and more abundant. We predicted a stress tolerance–productivity tradeoff [26,45,46], but there was no variation in stress tolerance. However, we did identify a possible productivity–abundance tradeoff where faster-growing plants were less abundant. We suspect this tradeoff was driven by a combination of variation in resource availability and competitive exclusion. While desiccation tolerance may allow *M. flabellifolia* to thrive in environments where other plants struggle, competitive exclusion may reduce population success in higher-resource environments. These findings also indicate that population abundance and plant size alone should not be used to guide harvest practices. Instead, local estimates of growth rate should be included in decision making. Finally, there are other aspects of the species’ biology and ecology that should be investigated in order to improve management and production outcomes. Future studies looking at seed development and germination, root biology, disease, and herbivory will help us build a more comprehensive understanding of this valuable species.

## Figures and Tables

**Table 1:** Correlation matrix of eight traits across the three major study sites. Pearson correlation values are shown with positively correlated traits shown in bule, and negatively correlated traits shown in red.

	No. of Inflorescences	Growth Rate	Leaves per cm	Leaf Area	Specific Leaf Area	Height	Relative Water Content	Percent Recovery
**No. of inflorescences**	1	−0.1423	0.084	−0.2768	−0.0377	−0.0179	0.0519	0.0079
**Growth rate**	−0.1423	1	−0.1667	0.069	0.0656	0.0032	−0.0054	0.1642
**Leaves per cm**	0.084	−0.1667	1	−0.3865	−0.1511	−0.3024	−0.0575	−0.064
**Leaf area**	−0.2768	0.069	−0.3865	1	0.0642	0.3211	−0.0823	0.0676
**Specific leaf area**	−0.0377	0.0656	−0.1511	0.0642	1	−0.0507	0.0343	0.0641
**Height**	−0.0179	0.0032	−0.3024	0.3211	−0.0507	1	−0.0203	0.0595
**Relative water content**	0.0519	−0.0054	−0.0575	−0.0823	0.0343	−0.0203	1	0.0488
**Percent recovery**	0.0079	0.1642	−0.064	0.0676	0.0641	0.0595	0.0488	1

## Data Availability

All data associated with this work is provided as supporting information (Table S1).

## Supplementary Materials

The following supporting information can be downloaded at https://www.mdpi.com/article/10.3390/plants11101332/s1, Table S1: Phenotypic data.
